# Insights Into the Role of Streptococcus mutans and Candida albicans in Dental Biofilm Formation and Cariogenicity: A Literature Review

**DOI:** 10.7759/cureus.86159

**Published:** 2025-06-16

**Authors:** Wanessa Fernandes Matias Regis, Francisco Ruliglésio Rocha, Ramille Araújo Lima, Beatriz Panariello, Simone Duarte, Anderson da Cunha Costa, Raimunda Sâmia Nogueira Brilhante, Lidiany Karla Azevedo Rodrigues

**Affiliations:** 1 Dentistry, School of Pharmacy, Dentistry, and Nursing, Universidade Federal do Ceará, Fortaleza, BRA; 2 Medical Microbiology, Universidade Federal do Ceará, Fortaleza, BRA; 3 Dentistry, Centro Universitário Christus, Fortaleza, BRA; 4 School of Dental Medicine, Lake Erie College of Osteopathic Medicine, Bradenton, USA; 5 Dentistry, University at Buffalo, Buffalo, USA

**Keywords:** candida albicans, caries progression, dental caries, microbiota, polysaccharides, review article, streptococcus mutans, virulence

## Abstract

Dental caries is a widespread chronic disease that affects a large proportion of both adults and children globally. *Streptococcus mutans* is widely recognized as the primary pathogen responsible for dental caries, while *Candida albicans* frequently coexists with it, often forming a synergistic relationship. Despite this, the specific virulence mechanisms of these microorganisms, both individually and in coaggregation, as well as their collective impact on cariogenic potential, remain incompletely understood. This comprehensive review aims to examine both original and review articles addressing the virulence characteristics of these species, both independently and in coaggregation, and to assess how these interactions contribute to tooth demineralization, polysaccharide production, and the expression of virulence genes. The research reviewed here provides valuable insights into the physiological interactions between the two species, showing that these interactions lead to increased acid production within their coexisting biofilm, which enhances the cariogenic potential. These insights could guide future studies aimed at developing targeted strategies for preventing or mitigating dental caries.

## Introduction and background

Dental caries is characterized by the demineralization of tooth enamel resulting from the metabolic activity of acid-producing bacteria within the dental biofilm. It is a dynamic and potentially reversible disease, influenced by the delicate balance between demineralization and remineralization processes [1], a balance often disrupted by frequent exposure to dietary fermentable carbohydrates [2]. Given that untreated caries in both permanent and deciduous teeth ranks among the most prevalent global health conditions [3], understanding the cariogenic microbiota and its pathogenic mechanisms is essential for the development of effective preventive and therapeutic strategies. Among these microbes, *Streptococcus mutans*, a Gram-positive bacterium, plays a central role due to its ability to produce extracellular polymeric substances (EPS) and organic acids from metabolized carbohydrates, particularly sucrose [4]. Its cariogenic potential is further enhanced by its acid tolerance, allowing it to thrive in low-pH environments and outcompete noncariogenic species within dental biofilm [4]. EPS production is critical for microbial adhesion, cell-to-cell aggregation, and the mechanical stability of the biofilm matrix [5]. To synthesize EPS, *S. mutans *secretes glucosyltransferases (GTFs), enzymes that hydrolyze sucrose, a disaccharide with an atypical glycosidic linkage involving glucose and fructose [6]. Although *S. mutans *is the most extensively studied cariogenic microorganism, other species, such as *Lactobacillus *spp., *Actinomyces *spp., *Streptococcus sanguinis*, and particularly the fungus *Candida albicans*, also contribute significantly to the initiation and progression of dental caries [1-7]. In this review, we emphasize the significance of *C. albicans *coaggregating with *S. mutans* and its emerging role in caries pathogenesis.

*C. albicans *exhibits multiple morphological forms, including yeast, pseudohyphae, and hyphae, with the latter being the most pathogenic [8]. Its hydrophobic cells exhibit strong adhesion to soft and hard tissues [9], and it secretes proteinases capable of degrading host proteins [10]. While *C. albicans *typically coexists harmlessly with the host, immune suppression can trigger its transition to a pathogenic state [11]. The virulence of *C. albicans *is attributed to its morphological plasticity, adhesin expression, biofilm formation, and the production of hydrolytic enzymes, which enable colonization and infection across multiple host sites [10]. Biofilm formation further enhances its resistance to antifungals and immune defenses [12], and microbial interactions within biofilms can be either cooperative or antagonistic, involving competition for nutrients or mutualistic exchanges of metabolites and growth stimulators [10]. In the oral cavity, co-adhesion between *C. albicans *and *S. mutans *has been linked to increased caries severity, particularly in early childhood caries, root caries, and recalcitrant infections [13-16]. Their combined pathogenicity is amplified by biofilm formation [17], where quorum sensing (QS), a density-dependent communication system, plays a critical role in regulating microbial behavior [18]. QS involves small signaling molecules called autoinducers, which accumulate with microbial growth and modulate gene expression [19]. In *C. albicans*, farnesol serves as the primary QS molecule, inhibiting the transition from yeast to hyphae, a key virulence factor [20]. At high concentrations, farnesol suppresses hyphal formation, tissue invasion, and even induces apoptosis, although it does not affect preexisting hyphae [12]. A second QS molecule, tyrosol (a derivative of tyrosine), has also been identified [21]. Unlike farnesol, tyrosol shortens the lag phase of fungal growth and promotes germ tube formation, accelerating hyphal development [22]. Thus, *C. albicans *morphogenesis is finely regulated by the opposing effects of tyrosol (stimulatory) and farnesol (inhibitory) [22].

This review examines the interactions between *S. mutans* and* C. albicans *in the pathogenesis of dental caries, with a focus on their combined effects on tooth demineralization, EPS production, and the regulation of virulence genes, both individually and in coaggregation. Relevant studies published within the last ten years were identified through systematic searches in Google Scholar, PubMed, and Scopus, using the following keywords: *Streptococcus mutans*, *Candida albicans*, tooth demineralization, polysaccharide production, expression of virulence genes, and dental caries. Articles were initially screened by title, followed by abstract review and full-text assessment. Only studies that specifically investigated the virulence mechanisms of these microorganisms and their interactions were included. To guide the selection process, we applied the PICO framework as follows: Population (P): In vitro and in vivo studies involving *S. mutans*, *C. albicans*, or both in coaggregation; Intervention (I): Evaluation of virulence traits, including biofilm formation, EPS production, gene expression, and demineralization capacity; Comparison (C): Analyses comparing the behavior of individual species versus their interactions; Outcome (O): Increased cariogenic potential, characterized by enhanced virulence factor expression and greater structural impact on dental tissues.

## Review

Exploring the virulence mechanisms of *S. mutans* and *C. albicans*

The *Streptococci *genus comprises spherical or oval Gram-positive bacteria (0.5-0.75 μm in diameter) that typically arrange in chains [23]. As facultative anaerobes of the phylum Firmicutes, most streptococcal strains are α-hemolytic or nonhemolytic [24]. These opportunistic pathogens are globally distributed, affecting populations across diverse ethnic, geographic, and socioeconomic backgrounds, underscoring their pandemic nature [25]. Its pathogenicity is largely influenced by the production of organic acids (e.g., lactic, formic, acetic, and propionic acids), which contribute to pH reduction and enhance biofilm virulence [26]. The acid environment promoted by *S. mutans* generates rapid pH modulations on the biofilm, which can drop the pH from neutral to acidic in less than 20 min [27]. The microorganisms’ ability to thrive in acidic environments results from the acid tolerance response, which is the capacity to adjust to acidic stress through previous exposure to low or sub-lethal pH levels [27].

The synthesis of exopolysaccharides is a critical virulence factor of *S. mutans*. These polysaccharides promote bacterial adhesion to tooth surfaces, including smooth ones, contribute to the structural integrity of biofilms, and modify their porosity. Such changes facilitate sugar diffusion within the biofilm matrix, leading to more pronounced pH reductions [28]. Acidification of the polysaccharide-rich matrix favors the growth of microorganisms that are tolerant to acid stress and promotes demineralization of dental enamel [26,29]. *S. mutans* produces three types of GTFs (Gtf*B*, Gtf*C*, and Gtf*D*) encoded by the *gtfB*, *gtfC*, and *gtfD *genes, respectively, whose cooperative action is essential for bacterial cell adherence with strong sucrose dependence [30]. These enzymes exhibit distinct functions: Gtf*B* produces water-insoluble glucans, Gtf*C* produces water-insoluble and water-soluble glucans, while Gtf*D* exclusively generates water-soluble glucans [28]. When excess sugar is available, *S. mutans *produces organic acids and extracellular matrix (ECM) and synthesizes intracellular glycogen-like polysaccharides that serve as carbohydrate reserves and help reduce osmotic stress [31].

*S. mutans *is classified into four serotypes (*c*, *e*, *f*, and *k*) based on the chemical composition of their cell surface rhamnose-glucose polymers [32]. These serotypes differ in their surface antigens, which enable bacterial adhesion to dental structures like enamel and dentin, thereby facilitating colonization [33]. The biosynthesis of rhamnose-glucose polysaccharide (RGP), a major cell wall component, is an important surface antigen that contributes to the bacterium’s structural identity and forms the basis for serotype classification [34]. Serotype variation arises from cell surface polysaccharide composition differences, which are mediated by enzymes like Rgp*G* [34]. Among these, serotype c is most prevalent in human populations [35], while serotypes *e*, *f*, and *k *show more restricted geographic distributions [36]. This genetic and structural diversity influences virulence traits, including oral surface adherence, biofilm formation, and interactions with other oral microbiota members [33].

Certain strains of *S. mutans *express collagen-binding proteins, such as Cnm and Cbm, which promote adhesion to collagen-rich surfaces like dentin [18]. In addition to contributing to colonization and pathogenicity in carious lesions, these proteins also modulate interspecies interactions, particularly with *C. albicans* [14].

*C. albicans* is a natural oral cavity resident that can transition from commensal to pathogenic under immunosuppression or host environment changes, leading to various infections [37]. First identified microscopically in thrush swabs by von Langenbeck in 1839 [38], *C. albicans *has since been recognized as a major pathogenic yeast in human infections. Its morphological versatility - existing as yeast, pseudohyphae, or hyphae - significantly contributes to pathogenesis, with hyphae enabling tissue penetration while yeast forms facilitate dissemination and less invasive infections [39].

*C. albicans *biofilm development occurs through four phases: adherence, initiation, maturation, and dispersal. During adherence, hydrophobic yeast-form planktonic cells attach to substrates, colonizing hard and mucosal surfaces [39]. The initiation phase sees microcolony formation, germ tube production, and early ECM synthesis. As biofilms mature, cells undergo morphological transitions that enhance ECM production [40]. This ECM comprises polysaccharides, proteins, extracellular DNA (eDNA), and other essential components that are crucial for the integrity of microorganisms and the resistance of biofilms. The accumulation of these components confers increased resistance to antimicrobial agents and host immune defenses [40].

**Figure 1:**
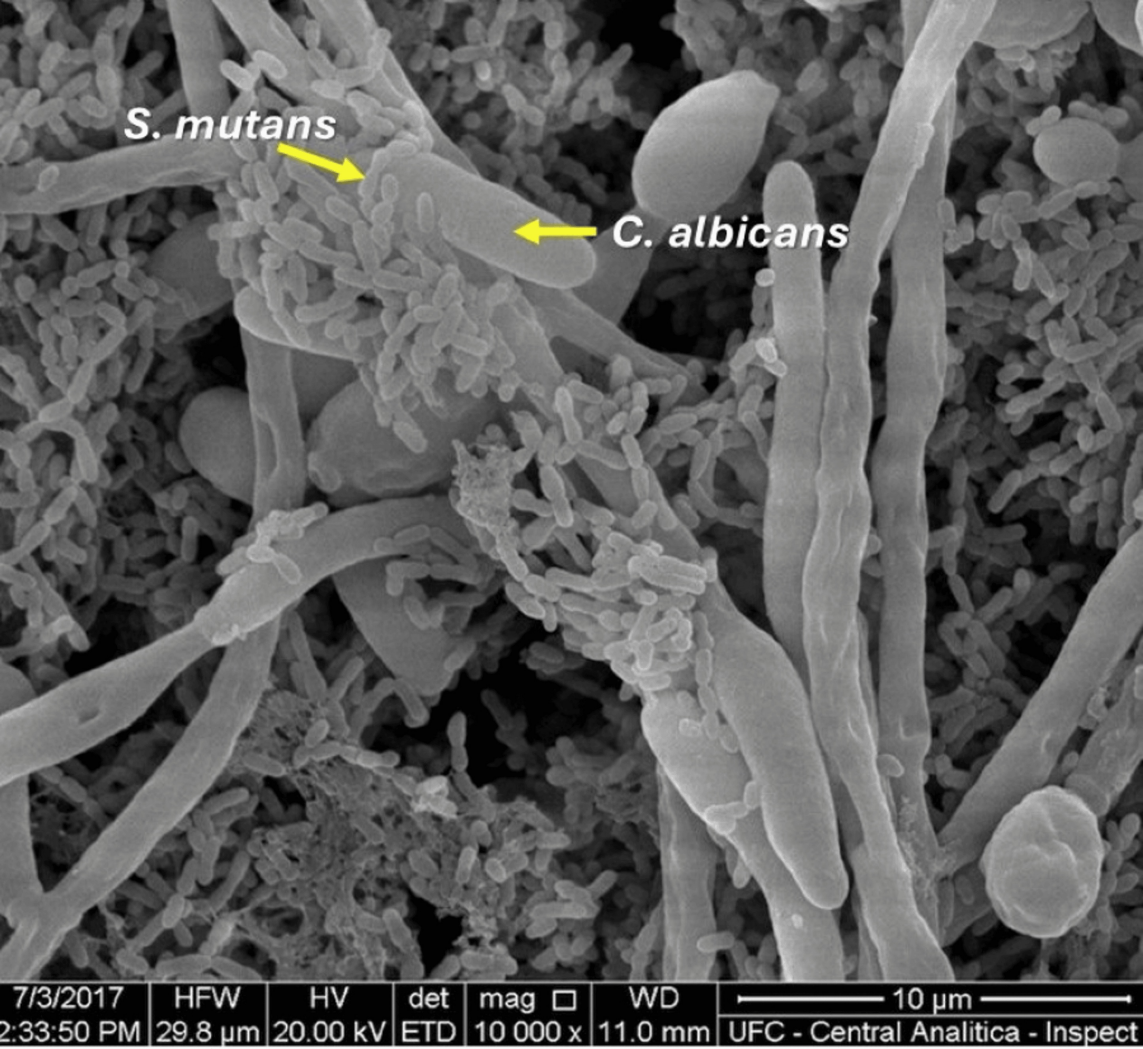
Scanning electron micrograph showing co-adhesion of Streptococcus mutans with Candida albicans Image acquired by Wanessa Fernandes Matias Regis

This biofilm-forming capacity and metabolic adaptability enable *C. albicans *to colonize diverse body regions and evade host immune responses [39]. In the oral cavity, *C. albicans *co-adhesion with bacteria is essential for biofilm colonization and persistence [41]. The fungus provides adhesion sites and growth-stimulating factors for bacteria, which in turn produce lactate that serves as a carbon source for yeast proliferation [42]. Figure 1 shows an image obtained via scanning electron microscopy, showing that *C. albicans *provides adhesion sites for *S. mutans*.

Synergistic interactions between *S. mutans *and *C. albicans *in biofilm acidogenesis and dental tissue demineralization

The development of dental caries is fundamentally linked to the production of acid within microbial biofilms. Carbohydrate fermentation by cariogenic microorganisms generates acids that lower biofilm pH [27], disrupting the demineralization-remineralization balance and leading to dental tissue breakdown when sustained over time [43]. Frequent sugar consumption exacerbates this process by creating a dysbiotic plaque environment, where acid-tolerant species, such as *S. mutans *and *C. albicans*, dominate [43].

*C. albicans*, an acidogenic and fermentative microorganism with cellular hydrophobicity, has been identified as a significant caries risk factor [13]. When coexisting with *S. mutans*, *C. albicans *increases sucrose metabolism and biofilm acidogenicity, rendering an acidic environment with a pH below 5.5 [13]. Although *C. albicans *lacks GTF enzymes for sucrose metabolism, it can metabolize fructose, glucose, and lactose to produce short-chain carboxylic acids, which reduce environmental pH [44], thereby facilitating hard tissue demineralization [45]. In contrast, *S. mutans *can hydrolyze sucrose into fructose and glucose, enabling cross-feeding interactions [46].

The cariogenic potential of these microorganisms is particularly concerning given *C. albicans*’ ability to colonize various dental surfaces, including enamel, dentin, and cementum, with penetration into dentin tubules [47]. Dual-species biofilms of *S. mutans *and *C. albicans *produce more numerous and severe smooth-surface lesions than single-species infections [48]. This enhanced virulence stems from several synergistic mechanisms: *S. mutans *excretes lactate, which serves as a carbon source for yeast growth while creating the low-oxygen tension preferred by *Streptococci *[41]. Additionally, the catalase produced by *C. albicans *improves *S. mutans*’ oxidative stress tolerance, promoting mutual survival within the biofilm [49].

Comparative studies demonstrate that dual-species biofilms cause greater enamel demineralization than *S. mutans *monocultures [13], with their synergistic interactions accelerating acid production and caries progression [43]. *C. albicans* plays a crucial role in biofilm maturation and maintaining the acidic environment necessary for enamel demineralization [13], although its cariogenic potential varies depending on strain characteristics and experimental conditions.

The ECM of dual-species biofilms exhibits significant differences from those of single-species communities. *C. albicans *presence increases EPS production, resulting in greater biomass and higher *S. mutans *cell counts [8]. The interaction between *C. albicans *cell wall components and *S. mutans*-produced EPS enhances biofilm robustness on both enamel and dentin, thereby increasing the potential for tissue demineralization [46].

**Figure 2:**
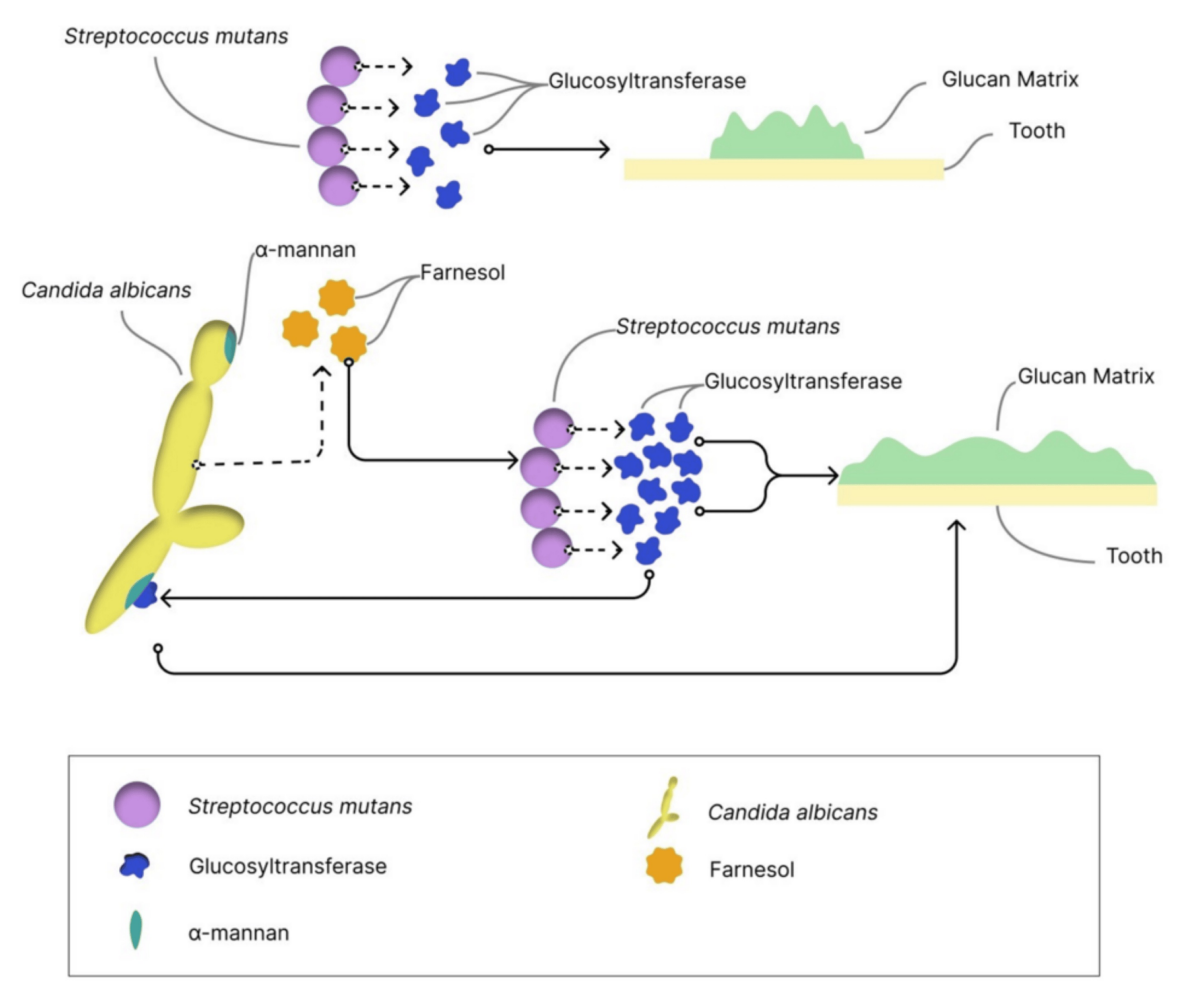
Overview of the key pathogenic mechanisms involved in the production of the glucan matrix by Streptococcus mutans and Candida albicans *S. mutans* synthesizes the glucan matrix through the activity of its Gtfs enzymes. The presence of *C. albicans *enhances this process through interaction between *C. albicans *α-mannan and *S. mutans *Gtfs, thereby promoting increased matrix formation. Fungal-derived farnesol can also upregulate gtfB expression, thereby enhancing glucan production and biofilm matrix formation. Gtf: glucosyltransferase Image created by Wanessa Fernandes Matias Regis

The glucan matrix, a key component of cariogenic biofilms, provides microbial adhesion sites, mechanical stability, and acidic microenvironments [50,51]. Both organisms contribute to this sticky matrix: *S. mutans *through GTF-mediated glucan synthesis from sucrose [31], and *C. albicans *through cell wall α-mannans that may facilitate Gtf binding and glucan matrix formation [52]. This protective matrix shields microorganisms from antimicrobial agents and host defenses [7]. Figure 2 provides an overview of the key pathogenic mechanisms employed by *S. mutans *and *C. albicans *in forming the glucan matrix.

The interaction between *C. albicans* and *S. mutans *appears to be highly context-dependent, with studies revealing synergistic and antagonistic dynamics. Sampaio et al. [53] demonstrated a synergistic relationship, showing *that C. albicans *enhances the cariogenic potential of *S. mutans *by increasing dentine demineralization within dual-species biofilms. Similarly, Klinke et al. [54] supported the cariogenic role of *C. albicans*, highlighting its capacity to produce acid from various carbohydrates, thereby contributing to the acidic microenvironment essential for tooth demineralization. In contrast, Dos Santos et al. [55] reported an antagonistic interaction in vivo, where secreted products from *S. mutans *inhibited *C. albicans *hyphal formation and reduced its virulence. Similarly, Barbosa et al. [56] suggest *S. mutans *has an attenuating effect on the virulence of *C. albicans*. These contrasting findings suggest that the outcome of *S. mutans*-*C. albicans *interactions may vary depending on environmental conditions, host context, and microbial composition, underscoring the complexity of their role in oral diseases.

The cariogenic potential of* S. mutans *is deeply rooted in its genomic repertoire, with complete genome sequencing of strains UA159, NN2025, LJ23, and GS-5 revealing key virulence determinants [57]. Among these, proteins harboring the LPXTG motif (leucine-proline-any amino acid-threonine-glycine) are critical for sortase-mediated anchoring to the bacterial cell wall, facilitating adhesion to tooth enamel, biofilm formation, and immune evasion [58,59]. Six conserved LPXTG-anchored proteins - Pac, FruA, DexA, GbpC, WapA, and WapE - further underscore the adaptability of *S. mutans *across different strains [56]. Major surface antigens, including Gtfs and glucan-binding proteins (Gbps), play pivotal roles in biofilm matrix assembly and caries pathogenesis [31,60].

The symbiotic relationship between bacteria and fungi within biofilms is mediated by extracellular signaling molecules and intercellular physical interactions, which facilitate the establishment and growth of cariogenic biofilms, accelerating caries development [61]. Notably, the co-aggregation of *S. mutans *and *C. albicans *enhances biofilm stability and acidogenicity, exacerbating caries progression. Frequent sugar consumption further amplifies acid production, while *C. albicans *secretes collagen-degrading enzymes under acidic conditions, intensifying biofilm virulence [46]. Acid production from carbohydrate fermentation by cariogenic microorganisms is a key driver of dental caries, as it reduces biofilm pH and disrupts the demineralization-remineralization equilibrium [27,43]. This acidogenic shift promotes a dysbiotic plaque environment, favoring the growth of acid-tolerant species. In the presence of *S. mutans* and *C. albicans*, enhanced acid production leads to a significant drop in biofilm pH, often to around 4.0-4.5 within 24 hours, which in turn promotes enamel demineralization and consequent calcium ion release from hydroxyapatite [41,62]. This synergistic interaction accelerates the cariogenic process.

Virulence gene expression and cross-kingdom interactions in dual-species cariogenic biofilms of *S. mutans *and *C. albicans*

*S. mutans* exhibits a symbiotic yet competitive relationship with *C. albicans *in mixed-species biofilms. GtfB binds strongly to α-mannan on the *C. albicans *cell surface, facilitating coaggregation and synergistic biofilm development [52]. This interaction is further modulated by fungal-derived farnesol, a QS molecule that, at high concentrations, disrupts *S. mutans *glycolytic activity and membrane permeability, reducing bacterial fitness [61]. Paradoxically, farnesol also upregulates gtfB expression, enhancing glucan production and biofilm matrix formation [44]. Moreover, *S. mutans *GtfB stimulates the expression of *C. albicans *adhesin genes (*hwp1*, *als1*, and *als3*), promoting fungal accumulation within biofilms [63].

Previous studies highlight the role of *S. mutans *membrane vesicles (MVs) in cross-kingdom interactions. MVs enriched with Gtfs integrate into *C. albicans *biofilms, boosting EPS production and upregulating fungal biofilm-related genes [64]. In contrast, MVs derived from ΔgtfBC mutants, which have the *gtfB *and *gtfC *genes deleted, fail to enhance biofilm formation, highlighting the crucial role of these genes in the biofilm development process [65].

The regulatory dynamics between these species are complex. The *S. mutans *VicRK two-component system, which plays a critical role in sensing environmental stress and regulating biofilm formation, is upregulated in the presence of *C. albicans*, resulting in increased EPS production and heightened cariogenic potential [66]. On the fungal side, *C. albicans *expresses Hwp1, a hyphal adhesin essential for epithelial attachment and biofilm stability, which interacts with *S. mutans *GtfB to enhance coaggregation [67,68]. Additional physical interactions, such as the binding of *S. mutans *to *C. albicans *hyphae via SspA/B proteins, further reinforce the structural and functional integrity of the mixed-species biofilm, with implications for increased tissue invasion and collagen degradation [44]. Tao et al. [69] demonstrated that *S. mutans *also modulates *C. albicans *morphology by secreting mutanocyclin, a tetramic acid compound that inhibits fungal filamentation via the Ras1-cAMP/PKA signaling pathway, specifically targeting the Tpk2 subunit. Their findings also identified key transcriptional regulators - *Sfl1*, *Ahr1*, *Nrg1*, and *Fcr1* - as critical mediators of this suppressive effect, highlighting the genetic basis of cross-kingdom antagonism. Moreover, the VicRK two-component system, conserved in both *S. mutans *and *C. albicans*, acts as a global regulator of stress responses, adhesion, and biofilm development [4,70]. In *C. albicans*, VicRK controls genes involved in hyphal formation and ECM production, further contributing to biofilm architecture and virulence [70]. Although hyphal structures are known to enhance biofilm integrity, their precise role in facilitating cross-kingdom interactions remains to be fully elucidated. In parallel, *S. mutans *expresses a group of surface proteins containing the LPXTG motif, a conserved amino acid sequence that enables anchoring of proteins to the bacterial cell wall. These anchored proteins, particularly GtfB, play a key role in enhancing biofilm cohesion and mediating physical interactions with *C. albicans *[33].

Key fungal genes, including *als1*, *als3*, and *hwp1*, contribute to adhesion and biofilm formation, with potential modulation by *S. mutans*-derived factors [68]. Farnesol sensitizes *S. mutans *to oxidative stress, downregulating virulence genes, while *C. albicans *upregulate *vicR *and *vicK *in* S. mutans*, enhancing EPS biosynthesis and cariogenicity [71]. The *hwp1 *gene further amplifies coaggregation through interactions with *GtfB.* Lu et al. [72] demonstrated that RNase III family coding genes in *S. mutans *significantly modulate dual-species biofilm development. Disruption of these genes altered the expression of QS and stress-response pathways, thereby attenuating biofilm formation. Feldman et al. [73] further characterized the transcriptional impact of pharmacological intervention using thiazolidinedione-8, a QS inhibitor targeting *C. albicans*. They observed altered expression of key virulence genes in both *C. albicans *and *S. mutans*, including downregulation of genes involved in adhesion (e.g., *ALS3*) and EPS production (*gtfB*), weakening the structural and cooperative integrity of the biofilm. In line with this, Bachtiar and Bachtiar [74] used qPCR to evaluate gene expression profiles in dual-species biofilms from clinical samples. They reported that co-culture conditions enhanced the expression of *S. mutans* gtfB and *gtfC*, along with increased transcription of *C. albicans *adhesion-associated genes such as *HWP1 *and *ALS1*. This upregulation reflects a coordinated transcriptional response that reinforces biofilm cohesion and virulence. Environmental context also influences gene expression.

Guo et al. [75] revealed that eDNA, a key structural component of the ECM, modulates biofilm development by influencing the transcription of biofilm- and stress-related genes in both organisms. Their study pointed to increased expression of *gtfB *and *gtfC *in the presence of eDNA, suggesting that extracellular cues contribute to transcriptional regulation and adaptive responses within the biofilm. Kim et al. [62] found that *C. albicans *strains deficient in genes involved in N- and O-linked mannan biosynthesis showed significantly reduced binding of *S. mutans *GtfB, impairing *S. mutans*’ ability to anchor its EPS onto *C. albicans*, weakening biofilm development, and highlighting the crucial role of gene-regulated receptor-ligand interactions in cross-kingdom synergy. Wu et al. [76] proteomic analysis revealed that 170 proteins in *C. albicans *were significantly altered upon exposure to *S. mutans *MVs, with 73 upregulated and 97 downregulated. Gene ontology and KEGG (Kyoto Encyclopedia of Genes and Genomes) pathway analyses indicated that these proteins primarily involve oxidation-reduction processes and galactose metabolism. Metabolomic profiling further confirmed that *S. mutans *MVs increased the expression of metabolites related to carbohydrate metabolism *in C. albicans*.

While several studies, including those by Ikono et al. [77] and Li et al. [78], evaluated the effects of antimicrobial agents such as nanochitosan and curcumin, these investigations did not extensively explore gene expression changes. However, their reduced biofilm biomass and microbial viability findings imply indirect transcriptional repression of genes associated with adhesion, EPS synthesis, and stress tolerance. The impact of probiotics on gene expression within cross-kingdom biofilms is also noteworthy. Bao et al. [79] and Huang et al. [80] demonstrated that *Lactobacillus plantarum *downregulates key virulence and adhesion genes in *S. mutans *and *C. albicans *in a dose-dependent manner. These observations suggest modulation of transcriptional responses as part of the biofilm-inhibitory mechanism.

These studies highlight the pivotal role of gene regulation in the formation, stability, and pathogenicity of *S. mutans*-*C. albicans *dual-species biofilms. Their coaggregation is orchestrated by a complex network of shared virulence factors, reciprocal gene regulation, and synergistic biofilm development, collectively enhancing their cariogenic potential. Although considerable progress has been made in unraveling these interactions, particularly involving GtfB, farnesol, and adhesins, further investigation is needed to fully define the molecular mechanisms underlying their pathogenic synergy.

Table 1 summarizes the virulence mechanisms of *S. mutans *and *C. albicans *in single-species and dual-species contexts, focusing on demineralization capacity, EPS production, and biofilm architecture.

**Table 1:** Summary of virulence mechanisms of SM and CA in single- and dual-species biofilms, focusing on demineralization, extracellular polysaccharides production, and biofilm architecture +: low production; ++: medium production; +++: high production CA: *Candida albicans*; Ins: insoluble polysaccharides; NA: not applicable; SM: *Streptococcus mutans*; Sol: soluble polysaccharides

Reference	Strain		Substrate	Demineralization						Extracellular polysaccharides						Biofilm Architecture		
																			SM	CA	SM		CA		SM+CA		SM		CA		SM+CA		SM	CA	SM+CA
	Surface	Inner		Surface	Inner	Surface	Inner	Sol	Ins	Sol	Ins	Sol	Ins																						
	Falsetta et al. (2014) [41]	UA159		SC5314	Sprague Dawley rats	++	NA	+	NA	+++	NA	++	++	+	+	+++	+++	++	+	+++
Eidt et al. (2019) [43]	UA159	ATCC 90028	Bovine enamel	+++	++	+	+	++	++	NA	NA	NA	NA	NA	NA	NA	NA	NA
Khoury et al. (2020) [51]	UA159	SC5314	Human enamel	NA	NA	NA	NA	NA	NA	++	++	+	+	+++	+++	++	+	+++
Sampaio et al. (2019) [53]	UA159	ATCC 90028	Bovine root dentine	++	NA	+	NA	+++	NA	++	++	+	+	+++	+++	+	+	++
Dos Santos et al. (2020) [55]	UA159	ATCC18804	96-well flat-bottom plates	NA	NA	NA	NA	NA	NA	NA	NA	NA	NA	NA	NA	++	++	+
Barbosa et al. (2016) [56]	UA159	ATCC 18804	In vitro	NA	NA	NA	NA	NA	NA	++	++	+	+	+++	+++	++	+	+++
Kim et al. (2021) [62]	UA159	SC5314	Hydroxyapatite discs	NA	NA	NA	NA	NA	NA	++	++	+	+	+++	+++	++	+	+++
Kim et al. (2021) [62]	UA159	SC5314	Human enamel	++	NA	NA	NA	+++	NA	NA	NA	NA	NA	NA	NA	NA	NA	NA
Ellepola et al. (2017) [63]	UA159	SC5314	96-well flat bottom plates	NA	NA	NA	NA	NA	NA	NA	NA	NA	NA	NA	NA	NA	++	+++
Wu et al. (2020) [64]	UA159	SC5314	Culture plates	NA	NA	NA	NA	NA	NA	NA	NA	NA	NA	NA	NA	NA	+	++
Yin et al. (2022) [65]	UA159	SC5314	96-well flat-bottom plates	NA	NA	NA	NA	NA	NA	NA	++	NA	NA	NA	+++	++	+	+++
Martorano-Fernandes et al. (2023) [68]	UA159	SC5314	96-well flat bottom plates	NA	NA	NA	NA	NA	NA	++	++	+	+	+++	+++	+	+	+++
Wu et al. (2022) [76]	UA159	SC5314	Bovine coronal dentine	NA	NA	+	NA	++	NA	NA	NA	NA	NA	NA	NA	NA	+	++
Ikono et al. (2019) [77]	ATCC 25175	ATCC 10231	96-well flat-bottom plates	NA	NA	NA	NA	NA	NA	NA	NA	NA	NA	NA	NA	++	+	+++

Table 2 summarizes the differential expression of key virulence genes in *S. mutans *and *C. albicans *during the formation of dual-species biofilms.

**Table 2:** Modulation of specific virulence genes that are upregulated or downregulated by SM and CA, along with their interactions +: low production; ++: medium production; +++: high production CA: *Candida albicans*; NA: not applicable; SM: *Streptococcus mutans*

Reference	Strain			Gene of virulence			
	SM	CA	Molecule	Upregulation		Downregulation	
				SM	SM	SM	SM
Dos Santos et al. (2020) [55]	UA159	ATCC18804	Supernatant of SM	NA	ywp1	NA	cph1, efg1, hwp1
Ellepola et al. (2017) [63]	UA159	SC5314	gtfB	NA	hwp1, als1, als3	NA	NA
Yin et al. (2022) [65]	UA159	SC5314	Caffeic acid phenethyl ester	NA	NA	gtfB, gtfC, gtfD	NA
Tao et al. (2017) [69]	UA159	SC5314	Mutanocyclin	NA	NA	NA	hwp1, ece1, flo8 tec1; ihd1, hyrt, rbt5, rhd3; csa1; rbe1; als; cht2, cht3, chs1
Lu et al. (2022) [72]	UA159	SC5314	NA	dcr1	ndt80, efg1, brg1	NA	NA
Feldman et al. (2016) [73]	UA159	SC5314	Thiazolidinedione-8	gtfB, gtfC, gtfD, gbpB, brpA, luxS, nox, sodA	NA	NA	hwp1, csh1, als3, sod1, sod2, cat1,
								Bachtiar and Bachtiar (2018) [74]	UA159	SC5314	NA	gtfB	NA	NA	NA
Guo et al. (2021) [75]	UA159	SC5314	eDNA	gtfC	spaP, eap1, hwp1, cdr2	NA	als3								
Li et al. (2019) [78]	UA159	SC5314	Curcumin	NA	NA	gtfB, gtfC, gbpB, comC, comD, comE	als1, als3
Bao et al. (2023) [79]	UA159	SC5314	Lactobacillus plantarum	atpD, eno	cht2	lacC, lacG	hwp1, ece
Huang et al. (2023) [80]	UA159	SC5314	Galacto-oligosaccharide	atpD, eno, lacC, lacG	NA	NA	hwp1, ece

## Conclusions

The synergistic relationship between *S. mutans *and *C. albicans *represents a critical pathogenic axis in developing dental caries, with GTFs and farnesol-mediated signaling emerging as promising therapeutic targets. While these molecular interactions offer compelling opportunities for disrupting biofilm integrity, substantial knowledge gaps remain in three key areas: (1) the precise mechanistic role of *C. albicans *in caries progression; (2) strain-specific differences in fungal virulence; and (3) the clinical relevance and translatability of current in vitro findings. Addressing these gaps will require innovative strategies, including creating advanced polymicrobial biofilm models that more accurately replicate the oral microenvironment and exploring combination therapies targeting both bacterial exopolysaccharide synthesis and fungal adhesion mechanisms. By closing these gaps, we can transform mechanistic insights into precision-based interventions for caries prevention.
